# Modulation of the transcription factor NF-κB in antigen-presenting cells by bovine respiratory syncytial virus small hydrophobic protein

**DOI:** 10.1099/jgv.0.000855

**Published:** 2017-07-17

**Authors:** Nicola Pollock, Geraldine Taylor, Fatoumatta Jobe, Efrain Guzman

**Affiliations:** ^1^​ School of Biosciences, Cardiff University, Cardiff, CF10 3AX, UK; ^2^​ The Pirbright Institute, Ash Road, Pirbright, Woking, RG8 0JU, UK

**Keywords:** respiratory syncytial virus, RSV, bovine, small hydrophobic, SH, NF-κB, dendritic cells

## Abstract

Bovine respiratory syncytial virus (BRSV) is an important cause of respiratory disease in young cattle and is closely related to human RSV (HRSV), which causes severe respiratory disease in infants and the elderly. The RSV genome encodes a small hydrophobic (SH) protein with viroporin activity. Previous studies have shown that recombinant BRSV lacking the SH gene (rBRSVΔSH) is attenuated in the lungs, but not in the upper respiratory tract, of calves and mucosal vaccination with rBRSVΔSH induced long-lasting protective immunity. Attenuation of rBRSVΔSH may be due to the ability of this virus to induce an early innate response as rBRSVΔSH induces higher levels of pro-inflammatory cytokines than wild-type (wt) rBRSV. In this study, we investigated the effects of the BRSV SH protein on NF-κB p65 phosphorylation, a master step in the regulation of pro-inflammatory cytokines. Expression of SH resulted in the inhibition of NF-κB p65 phosphorylation in response to BRSV infection and extracellular lipopolysaccharide, and a reduction in the production of pro-inflammatory cytokines. In contrast, rBRSVΔSH does not inhibit NF-κB p65 phosphorylation in bovine antigen-presenting cells, including monocytes, macrophages and dendritic cells, resulting in increased expression of pro-inflammatory cytokines and increased activation of T cells compared to cells infected with wt BRSV. These findings highlight an important role for the BRSV SH protein in immune modulation.

## Abbreviations

APC, Antigen presenting cells; BRSV, Bovine RSV; CHX, clycloheximide; DC, dendritic cells; LPS, Lipopolysaccharide; MDBK, Madin–Darby bovine kidney; m.o.i., multiplicity of infection; NF-κB, nuclear factor kappa-light-chain-enhancer of activated B cells; PIV, Parainfluenza virus; RSV, Respiratory syncytial virus; RANTES, regulated on activation, normal T cell expressed and secreted; SH, small hydrophobic; TAP1, Transporter associated with antigen processing 1; TLR, Toll-like receptors; TNF, Tumour necrosis factor.

## Introduction

Bovine respiratory syncytial virus (BRSV) is a major cause of respiratory disease in young calves [1] and is closely related to human RSV (HRSV), which is the single most important cause of bronchiolitis and pneumonia in children under 5 years of age [2]. HRSV and BRSV are genetically and antigenically similar, and the similarities in the epidemiology and pathogenesis of infection with these viruses indicate that comparative studies of their immunobiology will yield important insights that should benefit both man and cattle. BRSV and HRSV belong to the family *Pneumoviridae*, and contain a single-stranded, negative-sense RNA genome encoding 11 proteins: two non-structural proteins (NS1 and NS2); nucleocapsid (N) protein; phosphoprotein (P); matrix protein (M); three glycoproteins [small hydrophobic (SH), G (attachment) and F (fusion)]; M2-1 and M2-2, which control transcription and RNA replication; and the RNA polymerase (L).

Recombinant (r)BRSV in which the SH gene has been deleted (rBRSVΔSH) induces higher levels of inflammatory cytokines IL-1β and TNF-α than wild-type (wt) virus in Madin–Darby bovine kidney (MDBK) cells and bovine monocytes [3]. Similarly, rHRSVΔSH induces higher levels of IL-1β *in vitro* and IL-6 in the lungs of mice than wt HRSV [4]. The expression of many cytokines, including IL-1β and TNF-α, is regulated by the transcription factor NF-κB (nuclear factor kappa-light-chain-enhancer of activated B cells), which is activated by a series of phosphorylation and ubiquitinylation events resulting in phosphorylation of the p65 subunit and subsequent translocation to the nucleus where it helps to activate target gene transcription [5]. Antigen-presenting cells (APCs), including macrophages (Macs) and dendritic cells (DCs), are major sources of inflammatory cytokines, and NF-κB activation plays an important role in antigen presentation and T cell activation by APCs by regulating the level of expression of transporter associated with antigen processing 1 (TAP1) [6], major histocompatibility complex class I (MHC class I) [7] and co-stimulatory molecules such as CD40 and CD86 [8, 9].

Both BRSV and HRSV infect airway epithelial cells and induce an inflammatory response through the activation of networks of inflammatory and immunoregulatory genes. The exacerbated production of pro-inflammatory cytokines and chemokines in the airways in response to RSV contributes to bronchiolitis and pneumonia. The mechanisms involved in the induction and regulation of these immune modulators is not clearly defined. However, RSV activates the NF-κB complex, which is a major regulator of pro-inflammatory cytokine and chemokine genes. Other studies have demonstrated that the SH proteins of parainfluenza virus 5 (PIV-5), HRSV and human metapneumovirus (HMPV) play a role in NF-κB activation, inhibiting tumour necrosis factor alpha (TNF-α)-mediated signalling [10–12].

In order to understand the effects of the RSV SH protein on APC function and activation of inflammatory cytokines in APCs, we analysed the effects of BRSV SH protein expression on the phosphorylation of p65 and the expression of pro-inflammatory cytokines in bovine APCs. In addition, the effect of SH expression on antigen presentation and T cell activation was evaluated.

## Results

### Expression of SH inhibits phosphorylation of p65 in epithelial cells

**Fig. 1.**
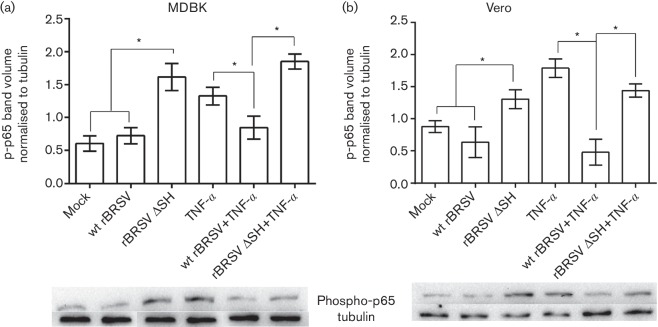
SH blocks phosphorylation of p65 in epithelial cells. MDBK (a) and Vero (b) cells were infected with recombinant virus (m.o.i. of 3) or mock-infected in the presence or absence of rTNF-α. Three hours post-infection, cell lysates were separated by sodium dodecyl sulfate polyacrylamide gel electrophoresis (SDS-PAGE) and levels of p65 phosphorylation determined by phospho-specific immunoblots. The graphs show phospho-p65 volumes normalized to tubulin volumes in the same sample. Columns indicate means of three replicates; error bars indicate standard deviation (sd); * indicates *P*<0.05. The blots shown are representative of three separate experiments.

We have previously shown that cells infected with rBRSVΔSH produced significantly greater amounts of pro-inflammatory cytokines IL-1β and TNF-α than those infected with the parental wt rBRSV [3]. To determine if these two viruses differentially modulate the NF-κB signalling pathway, MDBK and Vero cells were infected with rBRSV or rBRSVΔSH in the presence or absence or rTNF-α and phosphorylation of NF-κB p65 was determined by Western blot. At 3 h post infection, phosphorylation of p65 was not detected in cells infected with wt BRSV. In contrast, there was a significant increase in phospho-p65 in cells infected with rBRSVΔSH (Fig. 1). TNF-α increased phospho-p65 in both MDBK and Vero cells. However, the rTNF-α mediated increase in p65 phosphorylation of MDBK and Vero cells was inhibited in cells infected with wt BRSV but not in cells infected with rBRSVΔSH.

### Expression of SH inhibits phosphorylation of p65 in APCs

**Fig. 2.**
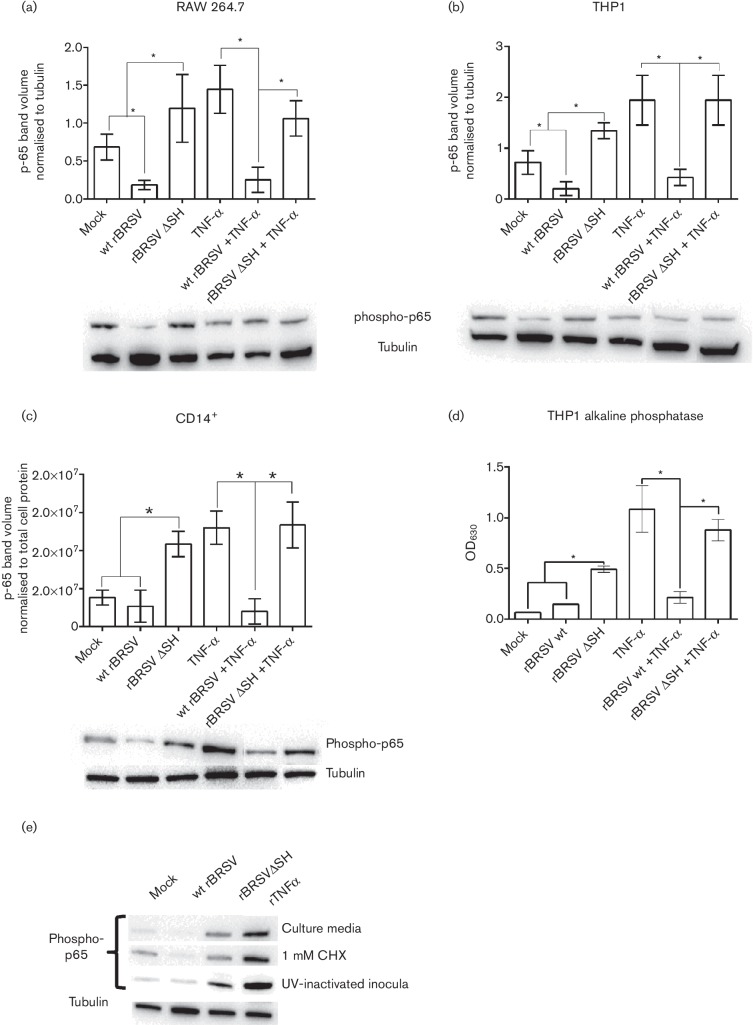
NF-κB p65 phosphorylation in APCs infected with rBRSV. RAW 264.7 (a), THP-1 (b) and bovine primary CD14^+^ cells (c) were infected with recombinant virus (m.o.i. of 3) or mock-infected in the presence or absence of rTNF-α. Three hours post-infection, cell lysates were separated by SDS-PAGE and levels of p65 phosphorylation determined by phospho-specific immunoblots. (a) and (b) Phospho-p65 volumes normalized to tubulin volumes in the same sample. (c) Phospho-p65 volumes normalized to total cell protein in each lane. (d) Alkaline phosphatase activity in culture supernatants from infected THP1-Blue NF-κB reporter cells. Columns indicate means of three replicates; error bars indicate standard deviation (sd); * indicates *P*<0.05. (e) NF-κB p65 phosphorylation in bovine primary CD14^+^ cells exposed for 3 h to Vero cell supernantant (mock), infected with wt BRSV, rBRSVΔSH (m.o.i. of 3), or treated with rTNF-α in: row 1, culture media; row 2, culture media containing 1 mM CHX; row 3, UV-inactivated inocula in culture media. All images are representative of three separate blots.

Mononuclear phagocytic APCs are a major source of inflammatory cytokines regulated by the NF-κB pathway. In order to determine if BRSV infection induced phosphorylation of p65 in APCs, the mouse macrophage cell line RAW 264.7 was infected with wt rBRSV or rBRSVΔSH in the presence or absence of rTNF-α and phosphorylation of NF-κB p65 was assessed by Western blot. As observed with epithelial cells, p65 phosphorylation was not detected in response to infection with wt BRSV. In contrast, there was a significant increase in phospho-p65 in cells infected with rBRSVΔSH. Infection with wt BRSV, but not infection with rBRSVΔSH, inhibited rTNF-α-mediated phosphorylation of p65 (Fig. 2a,b). The human monocytic cell line THP-1 was used to determine if the results obtained above were cell-specific. Infection of THP-1 cells with wt BRSV did not induce phosphorylation of p65 and wt rBRSV blocked TNF-α-mediated phosphorylation of p65 (Fig. 2c). In contrast, there was a significant increase in phospho-p65 in cells infected with rBRSVΔSH, and TNF-α-mediated induction of p65 phosphorylation was not inhibited by rBRSVΔSH. Since BRSV was being used in these studies, bovine primary CD14^+^ cells were infected with wt BRSV or rBRSVΔSH in the presence or absence of TNF-α. As observed for all other cells, infection of bovine CD14^+^ cells with wt BRSV did not induce phosphorylation of p65 and in fact blocked TNF-α-mediated phosphorylation of p65 (Fig. 2d). In contrast, there was a significant increase in phospho-p65 in bovine CD14^+^ cells infected with rBRSVΔSH, and TNFα-mediated induction of phospho-p65 was not inhibited by rBRSVΔSH. Infection of an alkaline phosphatase-producing THP-1 reporter cell line (THP1-Blue NF-κB), developed to measure activation of NF-κB, with rBRSVΔSH resulted in statistically higher levels of alkaline phosphatase compared to cells infected with wt rBRSV. Furthermore, wt rBRSV, but not rBRSVΔSH, inhibited the production of alkaline phosphatase by THP1-Blue cells exposed to TNF-α (Fig. 2e). To determine if incoming SH protein (i.e. within the virus particle) or SH protein induced after infection inhibits p65 phosphorylation, or if soluble mediators in the BRSVΔSH virus preparation were responsible for the increase in p65 phosphorylation, bovine CD14^+^ cells were infected as before in the presence of the translation inhibitor cycloheximide or were exposed to UV-inactivated virus. These studies demonstrated that phosphorylation of p65 was not inhibited by cycloheximide and that phosphorylation of p65 was even induced by UV-inactivated BRSVΔSH (Fig. 2e), suggesting that the increased level of p65 phosphorylation was due either to soluble mediators in the BRSVΔSH inoculum or that the SH protein in the wt BRSV inoculum taken up by CD14^+^ cells inhibits p65 phosphorylation.

**Fig. 3.**
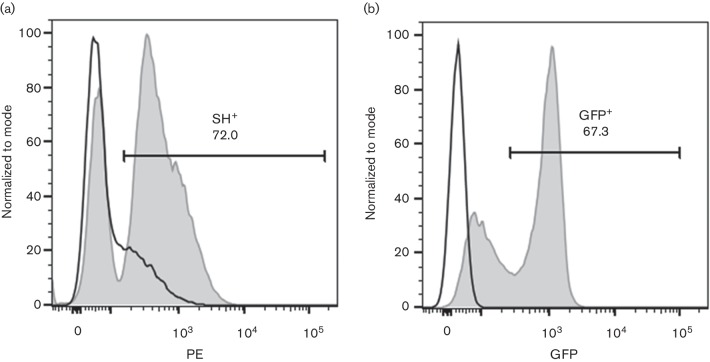
Expression of recombinant SH-V5/His_6_ in bovine CD14^+^. Primary bovine CD14^+^ cells were electroporated with pCDNA6 SH-V5/His6 (a) or pCDNA-GFP (b) or mock-electroporated (a and b). After 24 h in culture, cells were permeabilized and intracellular expression of SH-V5/His_6_ was detected using phycoerythrin (PE)-conjugated anti-V5; PE and GFP expression were measured by flow cytometry. Black histograms show autofluorescence of mock-electroporated cells stained with PE-conjugated anti-V5 (a) or autofluorescence alone (b); the grey-filled histogram shows fluorescence of electroporated cells stained with PE-conjugated anti-V5 (a) or GFP fluorescence (b). Figure representative of three experiments analysed in duplicate.

**Fig. 4.**
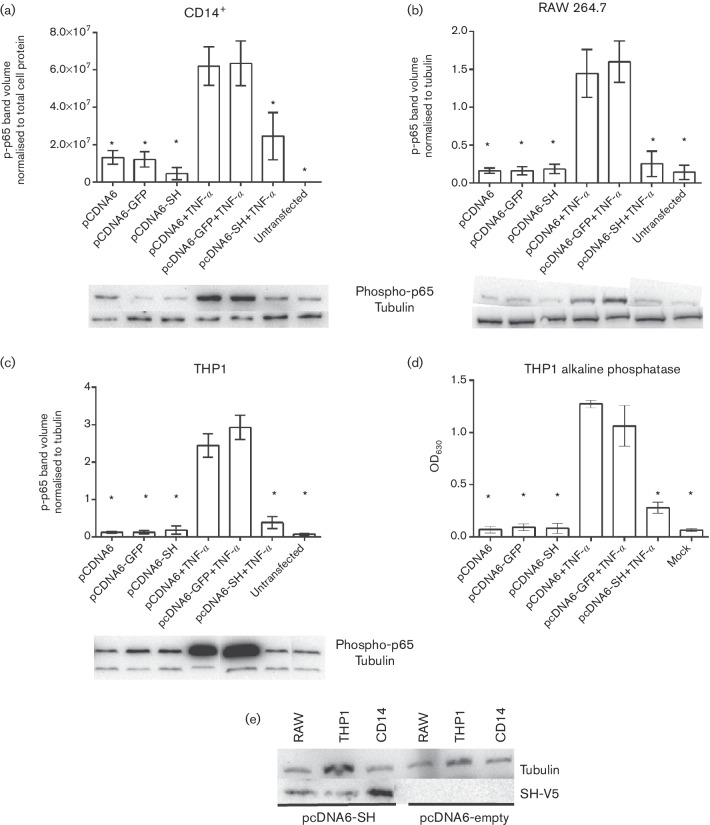
Inhibition of TNFα-induced NF-κB p65 phosphorylation in APCs transfected with plasmid-expressing BRSV SH protein. Bovine primary CD14^+^ cells (a), RAW 264.7 (b) and THP-1 (c) were electroporated with plasmid-expressing GFP or SH. Twenty-four hours later the cells were stimulated with rTNF-α as described in the Methods section. Cell lysates were separated by SDS-PAGE and levels of p65 phosphorylation determined by phospho-specific immunoblots. Blots are representative image of phospho-p65 specific immunoblots from electroporated cells. Phospho-p65 volumes in RAW 264.7 and THP-1 cells, respectively, normalized to actin volumes in the same sample. (d) Alkaline phosphatase activity in culture supernatants from transfected THP1-Blue NF-κB reporter cells. (e) Immunoblot showing expression of SH in transfected CD14^+^, RAW 264.7 and THP-1 cells 24 h post transfection. Columns indicate means of three replicates; error bars indicate standard deviation (sd); * indicate *P*<0.05. All images are representative of three separate blots.

In order to determine if expression of SH alone can inhibit phosphorylation of p65, RAW 264.7, THP1 and CD14^+^ cells were electroporated with a mammalian plasmid-expressing BRSV SH fused to V5 and hexa-histidine (his_6_) tags or a plasmid-expressing green fluorescent protein (GFP). Transfection efficiencies were approximately 70 % (Fig. 3a, b). Upon addition of extracellular rTNF-α, the cells transfected with GFP showed an increase in phosphorylated p65. In contrast, cells transfected with SH-V5/his_6_ did not show an increase in p65 phosphorylation (Fig. 4). These results indicate that the SH protein alone can inhibit p65 phosphorylation and suggest that incoming SH protein included in the wt BRS virions (inoculum) is responsible for blocking the NF-κB pathway at least during the first 3 h of infection. In addition, expression of the BRSV SH protein inhibits TNF-α-induced phosphorylation of the NF-κB p65 subunit.

### Wild-type BRSV inhibits degradation of IκBα

**Fig. 5.**
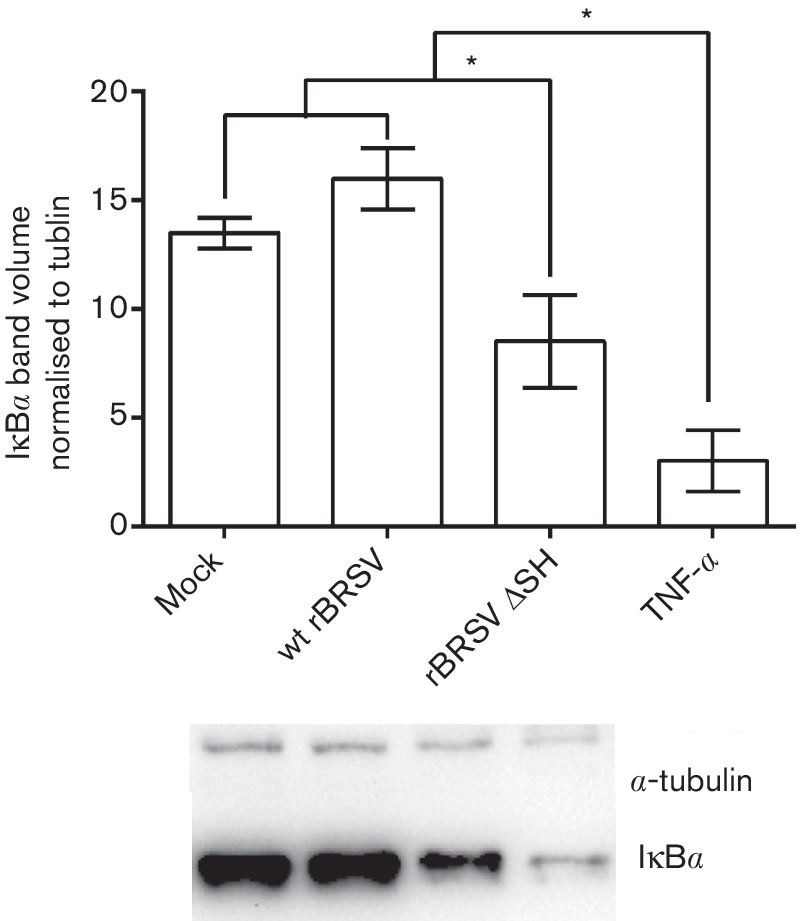
Wild-type rBRSV inhibits degradation of IκBα. Bovine primary CD14^+^ cells were mock-infected or infected with wt rBRSV or rBRSVΔSH (both m.o.i. of 3) for 3 h or treated with rboTNF-α for 10 min. Cell lysates were separated by SDS-PAGE and levels of IκBα determined by immunoblot. (a) Representative Western blot showing IkBα and endogenous α-tubulin. (b) Volumes of IκBα normalized to endogenous α-tubulin in each corresponding lane. Error bars indicate standard deviation (sd); * indicates *P*<0.05.

NF-κB/p65 transcription factors are present in the cytosol in an inactive state complexed with the inhibitory IκB proteins. Activation occurs via phosphorylation of IκBα followed by proteasome-mediated degradation that results in the release and nuclear translocation of active NF-κB. To determine how expression of SH by wt BRSV blocked activation of p65, primary bovine CD14^+^ cells were infected for 3 h with wt rBRSV, rBRSVΔSH at an m.o.i. of 3, mock-infected or activated with TNF-α for 10 min. Total cytosolic amounts of IκBα were determined by immunoblot. Fig. 5(a, b) shows that levels of cytosolic IκBα reduced after infection with rBRSVΔSH or treatment with TNF-α, whereas in wt BRSV-infected cells the levels of IκBα were not significantly different from those in mock-infected cells. These results indicate that inhibitory IκB proteins are not degraded in response to wt BRSV infection, resulting in inhibition of activation of p65.

### Expression of BRSV SH inhibits expression of cytokines

**Fig. 6.**
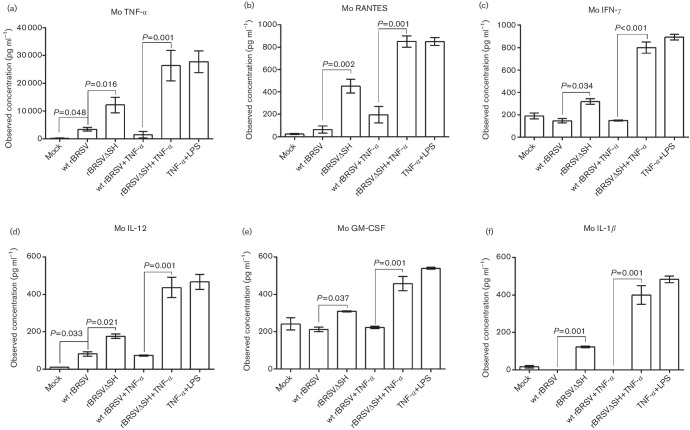
Cytokine production in mouse macrophages infected with rBRSV. RAW264.7 cells were infected with wt rBRSV, rBRSVΔSH or were mock-infected in the presence or absence of TNF-α for 16 h. Murine (Mo) cytokine production in culture supernatants was analysed by bead array ELISA. Columns represent means of three separate experiments analysed in duplicate. Error bars indicate standard error of the means.

To establish if SH-mediated inhibition of phospho-p65 expression resulted in decreased production of NF-κB-dependent cytokines, RAW 264.7 cells were infected with wt rBRSV or rBRSVΔSH in the presence or absence of TNF-α and cytokine expression in culture supernatants was analysed by multiplex ELISA, 24 h post infection. Expression of cytokines was significantly higher in cells infected with rBRSVΔSH compared to cells infected with wt rBRSV (Fig. 6). The addition of TNF-α significantly increased the production of cytokines. However, in cells infected with wt rBRSV and treated with TNF-α, cytokine expression was not significantly different from that in mock-infected cells (Fig. 6a–f).

**Fig. 7.**
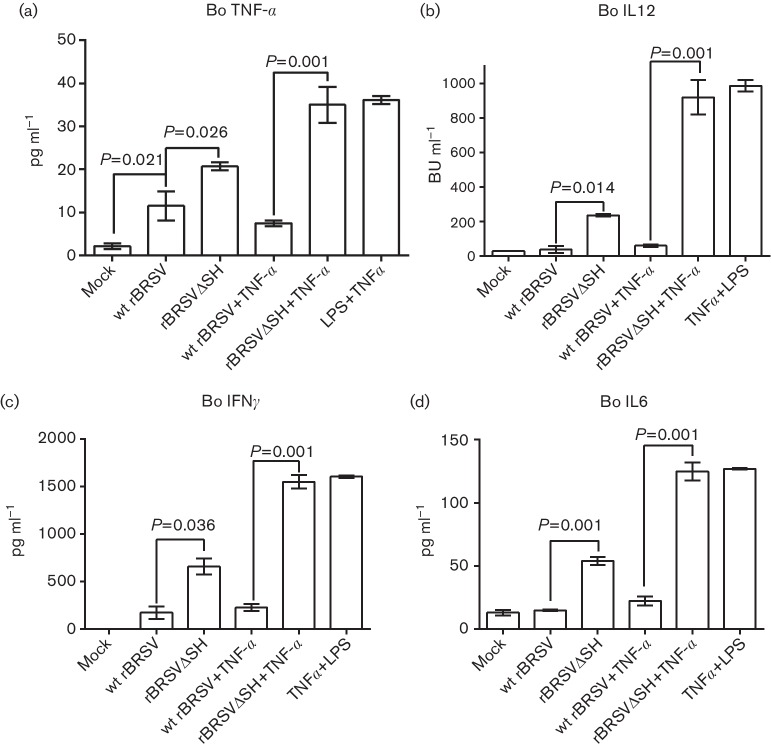
Cytokine production in bovine primary CD14^+^ cells infected with rBRSV. Bovine monocytes were infected with wt rBRSV, rBRSVΔSH or were mock-infected in the presence or absence of TNF-α. Bovine (Bo) cytokine production in culture supernatants was analysed by ELISA. Columns represent means of three separate experiments analysed in duplicate. Error bars indicate standard error of the means.

To confirm that these observations were not species-dependent, bovine primary CD14^+^ cells were infected with wt rBRSV or rBRSVΔSH in the presence or absence of TNF-α and cytokine production in culture supernatants was measured by ELISA, 24 h post infection. As observed in RAW 264.7 cells, supernatants from cells infected with rBRSVΔSH contained higher concentrations of cytokines compared to cells infected with wt rBRSV (Fig. 7). Furthermore, cytokine production in cells infected with wt rBRSV and treated with TNF-α was not significantly different from that in mock-infected cells (Fig. 7a–e).

### Bovine APCs expressing BRSV SH show reduced antigen presentation

**Fig. 8.**
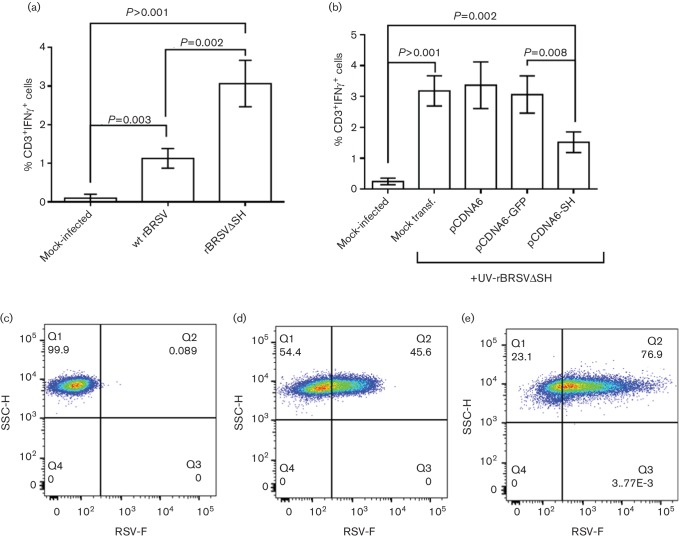
Expression of BRSV SH affects antigen presentation. Bovine primary CD14^+^ cells were (a) mock-infected or infected with wt rBRSV or rBRSVΔSH (both m.o.i. of 3) or (b) transfected with plasmids expressing SH or GFP and infected with UV-inactivated rBRSVΔSH. Activation of autologous T cells was detected by the expression of IFN-γ using flow cytometry. Bars indicate means of cells from four different animals analysed in duplicate. Error bars indicate standard error of the means. (c-e) Bovine primary CD14^+^ cells were mock-infected (c) or infected with an m.o.i. of 3 of wt BRSV (d) or rBRSVΔSH (e). Twenty-four hours later, the cells were fixed and stained with mouse anti-F. Dot plots are representative of cells from four different animals analysed in duplicate.

To determine if expression of SH affects antigen presentation and T cell activation, primary bovine CD14^+^ cells were mock-infected or infected with wt rBRSV or rBRSVΔSH, at an m.o.i. of 3, and cultured for 24 h with autologous CD3^+^ cells from calves infected 6 days previously with virulent, wt BRSV. Intracellular IFN-γ was measured by flow cytometry in CD3^+^ T cells. Peripheral blood mononuclear cells (PBMC) stimulated with APCs infected with wt rBRSV showed significantly lower frequencies of IFN-γ^+^ CD3^+^ T cells compared to cells stimulated with APCs infected with rBRSVΔSH (*P*=0.002; Fig. 8a). To confirm that expression of SH inhibited the ability of APCs to activate CD3^+^ T cells, primary bovine CD14^+^ cells were electroporated with pcDNA6-SH or control plasmids and then infected with UV-inactivated rBRSVΔSH and cultured for 24 h with autologous PBMC from calves infected with virulent wt BRSV. Samples transfected with pcDNA6-SH showed significantly lower frequencies of IFN-γ-producing cells compared to PBMC stimulated with APCs transfected with control plasmids (*P*=0.008 ; Fig. 8b). We then investigated the ability of wt or mutant viruses to differentially infect APCs. Primary bovine CD14^+^ cells were mock-infected or infected with wt rBRSV or rBRSVΔSH, at an m.o.i. of 3 p.f.u. per cell, and stained with a monoclonal antibody to the RSV F protein. Less than 50 % of cells infected with wt rBRSV expressed the F protein (Fig. 8c), whereas >75 % of cells infected with rBRSVΔSH expressed the F protein (Figs 8e and 7c). These results suggest that the increased ability of APCs infected with rBRSVΔSH to present antigen may be due, at least in part, to the greater uptake of virus compared to cells infected with wt rBRSV.

## Discussion

We have previously shown that BRSV lacking the SH gene exhibits site-specific attenuation in the bovine respiratory tract and that, despite reduced virus replication in the lungs, the mutant was as effective as wt BRSV in inducing protection from challenge with virulent BRSV 6 months after vaccination [3]. We also showed that rBRSVΔSH induced significantly higher levels of the pro-inflammatory cytokines IL1-β and TNF-α in bovine monocytes infected *in vitro*. We now show that expression of the BRSV SH protein in APCs reduces phosphorylation of NF-κB p65 affecting pro-inflammatory cytokine expression and antigen presentation.

The SH protein of BRSV is a 73–81 aa long, type II integral membrane protein with the C-terminus oriented extracellularly in the host and with a single predicted α-helical transmembrane domain [13]. The SH protein has been shown to form homopentameric ion channels activated at low pH [14] and permeabilize membranes functioning as viroporin [15, 16]. Various members of the family *Paramyxoviridae* encode SH proteins, for example simian virus 5 [17], parainfluenza virus 5 [11], mumps virus [10] and hMPV [12]. Indeed, one of the common features of mutant viruses lacking the SH protein is their capacity to induce NF-κB activation [10–12] in epithelial cell lines such as L929, Vero and MDBK. Although there is only 38 % amino acid identity between the SH proteins of BRSV and HRSV, both proteins appear to have similar functions. The SH domains responsible for the regulation of NF-κB remain to be elucidated.

We now show that mononuclear phagocytic APCs of human, mouse and bovine origin infected with rBRSV mutants lacking the SH gene phosphorylate the NF-κB p65 subunit more efficiently than APCs infected with wt rBRSV. In addition, APCs infected with wt rBRSV, but not those infected with rBRSVΔSH, were able to block phosphorylation of p65 following the addition of extracellular rTNF-α. Because we used 3 h infection during most of our assays, the increased phosphorylation of p65 in cells infected with rBRSVΔSH compared to wt virus may have been due to SH present within the incoming wt BRS virions (inoculum) or to increased levels of soluble mediators in the BRSVΔSH preparation. BRSV does not replicate in monophagocytic cells (data not shown and [18]), but it is possible for SH protein to be produced at later stages of the infection. Nevertheless, studies in which cells were transfected with the SH protein alone demonstrated that the SH protein plays an important role in inhibiting p65 phosphorylation. We also show that phosphorylation of p65 in wt BRSV-infected cells is due to the lack of degradation of IκBα, suggesting that SH interacts with IκBα, blocking either its ubiquitination or dissociation from the p65 subunit.

Using an NF-κB-alkaline phosphatase cell line, we also show that expression of SH results in reduced NF-κB activity. NF-κB must undergo a variety of posttranslational modifications, including phosphorylation or acetylation, to achieve its full biological activity. Inducible phosphorylation on distinct serine residues, including Ser276 and Ser536, has been shown to regulate NF-κB transcriptional activity without modification of nuclear translocation or DNA-binding affinity [19]. It has previously been shown that A549 cells overexpressing hMPV SH block phosphorylation of p65 in both Ser276 and Ser536 [12]. In the present study we only looked at phosphorylation of Ser536 and show that under all conditions and in all the cells used, TNF-α treatment significantly enhanced p65 phosphorylation which was inhibited by SH overexpression, further confirming the inhibitory role of the SH protein. The viruses used in these studies were produced in Vero cells that do not produce type I interferon, however it is possible for other cytokines produced during virus preparation to be responsible for the activation of the NF-κB pathway. Nevertheless, we present studies in which a plasmid-expressing SH was used demonstrate that the SH protein itself inhibits the NF-κB signal transduction pathway (Fig. 4d).

HRSV has been shown to regulate inflammatory responses in a variety of ways, including oxidative stress, Toll-like receptor (TLR) and inflammasome activation and IL-1β release [20]. In fact, there is evidence that the HRSV SH protein is important in activation of the inflammasome, since rHRSVΔSH failed to induce IL-1β secretion from primary human epithelial lung cells [21]. However, this observation contrasts with other studies in which both rBRSVΔSH and rHRSVΔSH induced higher levels of IL-1β than wt virus in a variety of different cells [3, 4]. Furthermore, there is evidence that virus proteins directly interact with elements of the NF-kB pathway [4].

APCs, including monocytes, macrophages and dendritic cells, are major producers of cytokines that in turn modulate downstream immune responses. RSV has been shown to interact with macrophages and dendritic cells (DC) and to affect their function. HRSV infects ~37 % of human monocytes and 35 % of alveolar macrophages, *in vitro* [22], and although BRSV does not replicate in bovine monocyte-derived DC (MoDC) [18], it does affect their viability and expression of MHC classes I, II, CD80 and CD86 [18]. We have shown that infection of mouse RAW 264.7 cells with wt rBRSV suppresses their capacity to express pro-inflammatory cytokines regulated by NF-κB compared to cells infected with rBRSVΔSH. Levels of RANTES (regulated on activation, normal T cell expressed and secreted), which is a T cell attractant that has been implicated in enhancing CD8^+^ cell function [23], are higher in rBRSVΔSH-infected RAW 264.7 cells than in cells infected with wt virus. This could result an increased number of T cells attracted to the area of infection, thereby enhancing the cell-mediated immune response to the virus. The relatively low levels of IL-12 observed in wt rBRSV-infected cells may explain T cell anergy [24] and the short duration of induced immunity following natural infection with wt HRSV. Moreover, lipopolysaccharide (LPS) and TNF-α–induced production of pro-inflammatory cytokines was reduced in cells infected with wt rBRSV but not in cells infected with rBRSVΔSH. Not only was this evident in immortalized monocytic cell lines, but also in bovine primary CD14^+^ cells, indicating the conserved nature of the function of SH across species. We have shown previously that bovine monocytes infected with rBRSVΔSH produce higher levels of TNF-α and IL1-β than cells infected with wt rBRSV [3]. Russell and colleagues have also shown that THP-1 and Hep-2 cells infected with rHRSVΔSH express higher levels of IL1-β than cells infected with wt HRSV [4]. In our studies, we observed that the levels of pro-inflammatory cytokines produced by rBRSVΔSH in RAW 264.7 cells and bovine CD14^+^ cells were significantly higher compared to levels produced by the wt virus. These results indicate that more than one cytokine is affected by the expression of the SH gene and a more comprehensive study of the genes regulated by SH is required.

It has previously been shown that BRSV affects bovine APC function by modulating cytokine transcription [18]. We now show that bovine APCs infected with wt rBRSV are hindered in their capacity to activate BRSV-specific T cells compared to APCs infected with rBRSVΔSH and that the SH protein is responsible for this effect. Although some studies have shown that HRSV infection of human MoDC does not affect their function [25, 26], other studies have shown that HRSV-infected cells have a reduced capacity to produce cytokines and present antigens to CD4^+^ T cells [24]. In addition, hMPV with deletions of both the G and SH proteins (ΔGSH) showed increased infectivity on human moDC [27] and we have also observed increased infectivity of bovine primary CD14^+^ by rBRSVΔSH compared to wt virus. This observed enhanced antigen presentation by rBRSVΔSH-infected APCs could be due to enhanced uptake as well as to an effect of the SH protein on cytokines/chemokines, MHC I-peptide formation or transport, co-stimulatory ligands expression or even as a result of SH pore formation on cellular membranes.

In our studies, all viruses were grown in Vero cells that do not produce type I IFN and we avoided the use of high-input m.o.i. of virus. Negative controls always contained mock-infected Vero supernatants to control for cellular components that could have non-specific effects on cell responses. Antigen presentation studies were performed using autologous cells and primary CD14^+^ cells were used *ex vivo* without the addition of extracellular cytokines such as GM-CSF and IL-4.

In summary, expression of the BRSV SH protein can affect the function of APCs by enhancing virus uptake and inhibiting the NF-κB transduction pathway, resulting in decreased pro-inflammatory cytokine production and decreased T cell activation.

## Methods

### Viruses and cells

Wild-type recombinant (r)BRSV and virus lacking the SH gene (rBRSVΔSH) were derived from full-length cDNA of BRSV strain A51908, variant Atue51908 (GenBank accession no. AF092942) [28]. Stocks of rBRSV were prepared in Vero cell monolayers, infected at an m.o.i. of between 0.1 and 0.5, in Dulbecco‘s modified Eagle’s medium (DMEM; Gibco-BRL) containing 2 % heat-inactivated foetal calf serum (FCS). All recombinant virus stocks were free from contamination with bovine viral diarrhoea virus (BVDV) and mycoplasmas. Virus titres were determined by plaque assay on Vero cell monolayers in six-well plates [29].

Bovine monocytes were prepared from heparinized venous blood by centrifugation at 1200 ***g*** over Histopaque 1086 (Sigma). The PBMCs were washed three times with PBS and CD14^+^ cells were purified by magnetic antibody cell sorting using anti-human CD14^+^ microbeads (Miltenyi Biotec) [30], following the manufacturer’s instructions. CD14^+^ cells were cultured in RPMI medium with 10 % FCS, ampicillin (0.1 mg ml^−1^) and 5×10^−5^ M 2-mercaptoethanol. Madin–Darby bovine kidney (MDBK) cells were cultured in Eagles minimal essential medium (EMEM) 10 % FCS, 100 U penicillin per ml and 100 mg streptomycin per ml. RAW 264.7 (mouse monocyte) cells were maintained in 10 % FCS DMEM, 100 U penicillin per ml, 100 mg streptomycin per ml and 5×10^−5^ M 2-mercaptoethanol. THP1 (human monocyte) cells were cultured in 10 % FCS RPMI media containing 100 U penicillin per ml, 100 µg streptomycin per ml and 5×10^−5^ M 2-mercaptoethanol. THP1-Blue NF-κB reporter cells (InvivoGen) were cultured in the presence of 10 µg ml^−1^ of blasticidin (InvivoGen). Unless otherwise indicated, all cells and media were obtained from The Pirbright Institute’s Central Services Unit.

### Infections and sample preparation

All infections were performed in the appropriate media containing 2 % FCS. For Western blots, 1×10^5^ cells of each cell type were infected at an m.o.i. of 3 of the corresponding virus for 3 h at 37 °C while being gently agitated; negative controls were cells cultured with mock-infected Vero cell culture lysate. Some cells were treated with 1 µg of bovine recombinant TNF-α [31]. Where indicated, cells were cultured with 1 mM cycloheximide (CHX, Sigma-Aldrich) for 3 h prior to infection and during the 3 h infection as described above. In some cases, viruses were UV-inactivated using a hand-held UV short-wave lamp (Fisher Scientific) at a distance of 2.5 cm for 5 min. Inactivation was confirmed by plaque assay as described above. All experiments were performed in triplicate. For cytokine ELISA, triplicate sets of RAW 264.7 and bovine CD14^+^ cells were infected as described above for 18 h and the supernatant collected.

Cell extracts were prepared from infected cells by centrifugation at 1500 ***g*** for 5 min. Cells were resuspended in 250 µl SDS sample buffer (Bio-Rad) containing complete mini-EDTA-free protease inhibitors (GE Healthcare) and denatured by boiling for 5 min.

### Cloning and expression of recombinant SH

The coding region of the rBRSV SH gene was amplified from BRSV Snook by reverse transcriptase-PCR (RT-PCR) using Invitrogen’s SuperScript III One-Step RT-PCR System with Platinum Taq High Fidelity kit following the manufacturer’s instructions. Hind*III* forward primer CTTAAGCTTATGAACAATACATC and EcoR*I* reverse primer CGAGAATTCGGTGCTTGATTGGT were used in the reactions; the underlined sections indicate the restriction sites. The PCR product was digested with Hind*III* and EcoR*I* and the resulting product ligated into the multiple cloning site pcDNA6 (Invitrogen) in frame with V5 and histidine tags using NEB’s Quick T4 DNA ligase and 2× Quick ligation buffer following the manufacturer’s instructions. The ligation reaction was transformed into NEB 5-α competent *E.coli* and grown on Luria–Bertani agar plates containing 100 µg ml^−1^ of ampicillin. Colonies were picked and plasmid DNA was extracted using Qiagen’s spin mini-prep kit. Plasmids were sequenced commercially by the Sanger method. A single clone with the exact nucleotide sequence of the SH gene and in frame with V5 and His tags was chosen and used in subsequent experiments.

### Transfections

Cells grown in six-well plates were transfected with endotoxin-free pcDNA6 SH-V5/His or the control plasmid pcDNA6-GFP (Invitrogen) using Lipofectamine 2000 Reagent (Invitrogen) following the manufacturer’s instructions. Twenty-four hours after transfection, the media were refreshed and, in some cases, 1 µg of LPS (K12, Invivogen) and/or rTNF-α were added to cells for 1 h. Cells were harvested and lysed into 150 µl of SDS sample buffer (Bio-Rad). Expression of the SH gene was confirmed by Western blot using mouse anti-V5 Tag (Serotec) and mouse anti-Histidine Tag (Serotec). Primary bovine CD14^+^ cells were electroporated in an Amaxa’s Nucleofector using a Human Monocyte Nucleofector Kit.

### Western blots

Samples were separated by SDS-PAGE and proteins transferred to polyvinylidene fluoride membranes (Bio-Rad) by semi-dry electroblot using Bio-Rad’s Trans-Blot Turbo Transfer System. The membranes were then blocked with 5 % dry milk in PBS for 1 h, washed thoroughly with 0.05 % Tween 20 in PBS (PBS-T), then incubated with the primary antibodies overnight at 4 °C: rabbit anti α-tubulin at 1 : 5000 (ab15246 Abcam); rabbit anti-phospho NF-κB p65 Ser536 (#3033, Cell Signalling) at 1 : 2000 diluted in 5 % dry milk in PBS; rabbit anti-IκBα (#9242, Cell Signalling). The membranes were washed five times with PBS-T and then incubated with goat anti-Rabbit IgG HRP (#7074, Cell Signalling) at 1 : 2000 dilution in 5 % dry milk in PBS for 1 h at room temperature. After thoroughly washing the membranes with PBS-T, Clarity Western ECL substrate (Bio-Rad) was prepared following the manufacturer’s instructions, added to the membranes and incubated for 5 min before photographing using Bio-Rad ChemiDoc MP imaging System. Data analysis was performed using ImageLab (Bio-Rad). For data generated using cell lines, band densities relative to the phosphorylated p65 band in the media-treated sample were quantified and corrected to the density of the tubulin band present in the same sample. For primary bovine CD14^+^ cells, Mini-PROTEAN TGX Stain-Free Precast Gels (Bio-Rad) were used to quantify the total protein content of each lane using the Image Lab (Bio-Rad) and the band volumes of phospho-p65 were normalised to the total protein content present in each lane.

### Enzyme-linked immunosorbent assay (ELISA)

Expression of the bovine cytokines TNF-α, IL-12, IL-6 and IFN-γ was detected by ELISA as previously described [32–36] using the supernatants from primary bovine CD14^+^ cells, 18 h post infection. Supernatants from RAW 264.7 cells taken 18 h post infection were used to measure cytokines using Bio-Rad’s Bio-Plex 200 Mouse Bio-Plex Pro Cytokine Assay following the manufacturer’s instructions.

### NF-κB reporter assay

THP1-Blue NF-κB cells were infected as described above and cultured for 24 h at 37 °C. Then 20 µl of culture supernatant from each well were mixed with 200 µl of QUANTI-Blue alkaline phosphatase detection medium (InvivoGen), following the manufacturer’s instructions, in a 96-well flat bottomed Maxi-Sorb (Nunc) ELISA plate and incubated for 4 h at 37 °C. Absorbance at 630 nm was measured on an ELx808 ELISA reader (BioTek).

### Antigen presentation

PBMCs from calves inoculated intranasally and intratracheally with 10^4^ p.f.u. BRSV, Snook strain, as described previously [37] were collected 6 days post infection. CD14^+^ and CD3^+^ cells were separated by Macs. as described above. and rested overnight in culture media at 37 °C. CD14^+^ cells were then infected with wt BRSV or rBRSVΔSH, or transfected with plasmids expressing SH or control plasmids. as described above. and incubated with UV-inactivated BRSV (m.o.i. of 3). 1×10^5^ CD14^+^ cells were plated into each well of a 96-well U-bottomed plate (BD Biosciences) and mixed with autologous CD3^+^ T cells at a ratio of 5 CD3^+^ to 1 CD14^+^ cell; Brefeldin A (10 µg ml^−1^, Sigma) was added to each well. The plate was vortexed briefly and incubated for 4–6 h at 37 °C. Live T cells were stained with Near-IR Live/Dead Stain (Life Technologies) and mouse anti-bovine CD3 (clone MM1A, VMRD) conjugated to allophycocyanin (APC). Cells were fixed and permeabilized using BD Biosciences’ Cytoperm/Cytofix buffer following the manufacturer’s instructions. Intracellular IFN-γ was detected using mouse anti-bovine IFN-γ conjugated to phycoerythrin (PE; clone CC302, Serotec). A minimum of 50 000 live events were acquired in a BD LSRFortessa. Single/live events were analysed using FlowJo version 10.7.

### Flow cytometry of virus-infected cells

Primary bovine CD14^+^ cells were infected at an m.o.i. of 3 p.f.u. per cell with recombinant wt and mutant virus in 96-well U-bottomed plates for 90 min in the presence of 100 µl of RPMI 1640. Then, the cells were washed twice and resuspended in 100 µl of RPMI containing 10 % FCS and cultured for 24 h at 37 °C. The cells were then washed once with PBS and stained with mouse anti-RSV F clone mAb19 [36] and Live/Dead Aqua for 10 min, washed twice with PBS, fixed with Cytofix/Perm buffer (BD) for 10 min, washed three times, stained with anti-mouse-AlexaFluor 647 for 10 min and washed as before. After the last wash, the cells were resuspended in 500 µl of PBS and analysed in a BD LSRFortessa as described above.

### Statistical analysis

Parametric one way analysis of variance (ANOVA) and Tukey’s multiple comparison tests were performed on all the data sets using GraphPad Prism 6.
